# *EHBP1*, *TUBB*, and *WWOX* SNPs, Gene-Gene and Gene-Environment Interactions on Coronary Artery Disease and Ischemic Stroke

**DOI:** 10.3389/fgene.2022.843661

**Published:** 2022-04-26

**Authors:** Chun-Xiao Liu, Rui-Xing Yin, Xiao-Li Cao, Zong-Hu Shi, Feng Huang, Bi-Liu Wei, Guo-Xiong Deng, Peng-Fei Zheng, Yao-Zong Guan

**Affiliations:** ^1^ Department of Cardiology, Institute of Cardiovascular Diseases, The First Affiliated Hospital, Guangxi Medical University, Nanning, China; ^2^ Department of Neurology, The First Affiliated Hospital, Guangxi Medical University, Nanning, China; ^3^ Department of Prevention and Health Care, The Fourth Affiliated Hospital, Guangxi Medical University, Liuzhou, China

**Keywords:** EH domain-binding protein 1, tubulin beta class I, WW domaincontaining oxidoreductase, single nucleotide polymorphism, coronary artery disease, ischemic stroke

## Abstract

The associations among the EH domain-binding protein 1 (*EHBP1*), tubulin beta class I (*TUBB*), and WW domain-containing oxidoreductase (*WWOX*) single nucleotide polymorphisms (SNPs) and coronary artery disease (CAD) and ischemic stroke (IS) are not yet understood. This study aimed to detect the associations of these SNPs, gene-gene and gene-environment interactions and CAD and IS in the Guangxi Han population. A total of 1853 unrelated subjects were recruited into normal control (*n* = 638), CAD (*n* = 622), and IS (*n* = 593) groups. Related genotypes were determined by high-throughput sequencing. The genotypic and minor allelic frequencies of rs2278075 were different between the CAD and control groups, and those of rs2710642, rs3130685, and rs2278075 were also different between the IS and control groups. The rs2278075T allele, rs3130685-rs2222896-rs2278075, rs3130685-rs2222896-diabetes, rs3130685-rs2222896-drinking, and haplotype rs2710642A-rs10496099C-diabetes interactions were associated with increased risk, while G-T-G-C-G-A and G-T-T-T-G-T-drinking were associated with reduced risk of CAD. The rs2278075T and rs2710642G alleles, rs2710642G-rs10496099C haplotype, rs3130685-rs2278075-rs2222896, and rs2710642-rs2278075-hypertension interactions aggravated the association with IS, whereas the rs3130685T allele, rs2710642A-rs10496099C haplotype and the interactions of H1 (s2710642A-rs10496099C)-H2 (rs2710642G-rs10496099C)-drinking and I1 (A-C-G-C-A-A)-I3 (A-C-G-T-A-A)-I4 (A-C-G-T-G-A)-I5 (G-T-G-C-G-A) diminished the association with IS. Carrying *WWOX* rs2278075T was strongly associated with CAD or IS, while *EHBP1* rs2710642 and *TUBB* rs3130685 might alter the association of IS by modifying the serum lipid profile. This study demonstrates that the *EHBP1*, *TUBB*, and *WWOX* SNPs, gene-gene and gene-environment interactions are associated with the risk of CAD and IS in the Guangxi Han population.

## Introduction

Cardiovascular disease (CVD) including cerebrovascular disease is the leading causes of death worldwide. In 2015, the global mortality of CVD was approximately 32.1%, of which coronary artery disease (CAD) accounted for 49.8% and ischemic stroke (IS) accounted for 68% (Arsava et al., 2017; Bede et al., 2020). Dyslipidemia is the key trigger of atherosclerosis, leading to odds ratios of CAD and IS of 3.25 and 1.84, respectively. However, dyslipidemia is also a controllable risk factor (Bede et al., 2020).

Dyslipidemia is genetically susceptible. The difference in lipid-related gene expression determines the diversity of serum lipid phenotypes. Several studies have reported associations between the SNPs in the EH domain-binding protein 1 gene (*EHBP1*) (Colafella and Denton, 2018), tubulin beta class I gene (*TUBB*), and WW domain-containing oxidoreductase gene (*WWOX*) (Denis et al., 2011; Deng et al., 2020) and dyslipidemia (GBD 2016 Disease and Injury Incidence and Prevalence Collaborators, 2017; Diener and Hankey, 2020), atherosclerosis and CAD. The mechanisms involved in lipid metabolism for these genes have been described. *EHBP1* regulates vesicle transport by its encoded protein, binds endocytic vesicles to the actin cytoskeleton, and plays a core role in modulating glucose transporter 4 in adipocyte transport (Fan et al., 2019). *EHBP1* is an effector molecule for oncogene family member 8 (Rab8) and Rab10. Rab10-EHBP1-EHD2 forms a trimer complex that plays a critical role in hepatocyte lipid phagocytosis and the autophagic digestion of lipid droplets (Fuchs and Whelton, 2020). The proprotein convertase subtilisin/kexin type 9 gene (*PCSK9*) reduces the recycling of low-density lipoprotein receptors (LDLRs) and redirects LDLRs to late endosomes and lysosomes for degradation, facilitating an increase in serum lipids. A study found that the expression of the EHBP1 protein was downregulated in cells overexpressing *PCSK9*. This revealed the existence of the *EHBP1* accompanying pathway linked to lipid metabolism (Gabb et al., 2016). Moreover, SNPs of the rs2710642 and rs10496099 in the *EHBP1* were correlated with low-density lipoprotein cholesterol (LDL-C) (Giagtzoglou et al., 2013) and atherosclerosis (Golan et al., 2019), respectively. The *TUBB* encodes β-tubulin. Mutations in *TUBB* cause microtubule damage, resulting in complex diseases (Denis et al., 2011). A genome-wide association study (GWAS) analyzed the susceptibility to genetic variants of early-onset hyperlipidemia in 8,073 Japanese individuals (age ≤65 years) and found that the SNPs of the rs3132584 and rs3130685 in the *TUBB* were associated with serum LDL-C levels (Guilherme et al., 2004). *WWOX* encodes a 414 amino acid protein that is involved in a variety of important cellular processes, including steroid metabolism. Studies have demonstrated that *WWOX* plays an important physiological role in lipid and lipoprotein metabolism in mouse and human genetic models. It is mediated through the ABCA1/apolipoprotein (Apo) A1 pathway, and it binds to a series of potential ligands of proline-rich PPxY motifs and other kinds of cell proteins. It is also involved in a variety of other pathways, including cholesterol homeostasis, fatty acid biosynthesis and triglyceride (TG) metabolism (Iatan et al., 2014; Hasan et al., 2020), GWAS indicated that there was an association between the fasting lipid profile of 1,087 participants in an offspring cohort (mean age 47 years, 52% female) and *WWOX*. The correlation between the *WWOX* rs2222896AA genotype and LDL-C was two times that of the rs2222896AG or rs2222896 GG genotypes (Johannsen et al., 2018). Additionally, the *WWOX* rs2278075 SNP was associated with CVD (*p* = 5.0 × 10^−9^) (Joseph et al., 2017). In a previous study in Guangxi Maonan population, we revealed that the *EHBP1* rs2710642 and rs10496099, *TUBB* rs3132584 and rs3130685, and *WWOX* rs2222896 SNPs interacted with several environmental factors to modify blood lipid profiles (Kathiresan et al., 2007; Liu et al., 2020). However, the association between these genes and the risk of CAD or IS in Chinese populations is unclear.

This study investigated the genotypes of SNPs (rs2710642, rs10496099, rs3132584, rs3130685, rs2222896, and rs2278075) in *EHBP1*, *TUBB*, and *WWOX* in the Guangxi Han population and analyzed the relationship between SNPs, gene-gene (G × G) and gene-environment (G × E) interactions, and the risk of CAD and IS, to explore the possible pathogenic mechanism of these genes and provides a theoretical basis for early CAD or IS prevention and treatment.

## Materials and Methods

### Study Population

The subjects of our cohort were inpatients and health physical examiners in the First Affiliated Hospital of Guangxi Medical University from September 2009 to December 2011. Data were listed in the database of our study team project, including their demographic characteristics, socioeconomic status, medical history, lifestyle factors, blood samples, and laboratory results. This study was conducted in accordance with the Declaration of Helsinki (2008 revised edition) and approved by the Ethics Committee of the First Affiliated Hospital of Guangxi Medical University. Informed consent was obtained from all subjects.

A total of 622 patients in the CAD group were diagnosed according to the following criteria: ① typical clinical manifestations, myocardial enzymes, and electrocardiogram changes; ② coronary angiography examination revealing at least one major coronary artery (right coronary artery, left circumflex, or left anterior descending artery) stenosis ≥50%; and ③ no cerebrovascular disease, valvular heart disease, congenital heart disease, or aneurysm. A total of 593 patients in the IS group were diagnosed according to the following findings: ① large atherosclerotic stroke and/or small-artery occlusive stroke (Khawaled et al., 2020); and ② no CAD, hemorrhagic cerebral infarction, transient ischemic attack, and cerebral embolism. A total of 638 normal subjects without a history of CAD and IS were used as a control group.

All participants were Han population and unrelated individuals whose ancestors lived in Guangxi for at least three generations. The ages of the subjects ranged from 18 to 80 years. Mean age and sex ratio of the three groups were matched (*p* > 0.05). The mean age and percentage of women in the control, CAD and IS groups were 62.07 ± 12.19 years, 26.5%; 62.34 ± 10.50 years, 27.8%; and 62.85 ± 12.17 years, 27.0%, respectively. They had no evidence of liver or kidney disease, autoimmune diseases, hematological diseases, tumors, severe infections, metabolic diseases (except diabetes), or mental diseases.

### Metabolic Parameters

A peripheral venous blood specimen (5 ml) was collected in the morning after fasting for more than 8 h. Serum lipid parameters were determined by an automatic analyzer (Model 7170A, Hitachi Co., Ltd., Tokyo, Japan). Serum LDL-C, total cholesterol (TC), TG and high-density lipoprotein cholesterol (HDL-C) were measured by commercially available enzyme assay kits, and serum ApoA1 and ApoB were measured by a turbidimetric immunoassay.

Dyslipidemia is defined as elevated levels of TC, TG or LDL-C or decreased HDL-C levels (Larsson et al., 2016). The reference ranges of serum TC, TG, HDL-C, LDL-C, ApoA1, and ApoB levels and the ApoA1/ApoB ratio in our hospital’s laboratory were 3.10–5.17, 0.56–1.70, 0.90–1.81, 2.70–3.10 mmol/L, 1.00–1.78, 0.63–1.14 g/L, and 1.00–2.50, respectively. Therefore, in this study, dyslipidemia was delimited by any of the following either alone or in combination: TC > 5.17, TG > 1.7, LDL-C > 3.10, and/or HDL-C < 0.9 mmol/L. Subjects with systolic blood pressure (SBP) ≥ 140 mmHg and/or diastolic blood pressure (DBP) ≥ 90 mmHg were defined as having hypertension (Leitwein et al., 2020). Body mass index (BMI) was calculated as weight (kg) divided by height (m) squared. Overweight and obese categories in China are defined as BMI >24 kg/m^2^ and >28 kg/m^2^, respectively (Levy et al., 2007).

### Genotyping

The basic principles of gene and SNP selection were as follows: ① *EHBP1*, *TUBB*, and *WWOX* were derived from GWAS associated with lipid metabolism. ② SNPs linked to lipid metabolism were selected by Haploview (Broad Institute of MIT and Harvard, Cambridge, MA, United States version 4.2). ③ Information on each SNP came from NCBI dbSNP Build 132 (http://www.ncbi.nlm.nih.gov/snp/). ④ SNPs with minor allele frequencies (MAFs) > 10% were included. ⑤ Associations between these SNPs (rs2710642, rs10496099, rs3132584, rs3130685, rs2222896, and rs2278075) and lipid metabolism or atherosclerotic cardiovascular disease in European and American populations were identified by previous studies.

DNA from peripheral blood samples was extracted using the phenol-chloroform method and stored at −80°C. Approximately 10 μL (10 ng/μL) of each sample was sent to the Department for Next-Generation Sequencing, Sangon Biotech Co., Ltd. (Shanghai, China) for genotyping. The absorbance of DNA samples met a ratio of A260 nm/A280 nm = 1.8 *via* a Shimadzu UV-1601 spectrophotometer, indicating that the DNA sample was pure. A HiSeqXTen sequencer (Illumina, San Diego, CA) was employed for SNP genotyping.

### Statistical Analysis

Statistical analysis was performed using SPSS software version 25 (SPSS Inc., Chicago, Illinois, United States). Normally distributed data are presented as the means ± standard deviation, and differences among groups were analyzed by Student’s unpaired *t*-test (the control *vs.* CAD groups, the control *vs.* IS groups) or one-way analysis of variance (associations between three genotypes and lipid levels). Nonnormally distributed data are shown as interquartile ranges and medians, and differences in groups were determined by Mann-Whitney nonparametric tests. Differences in qualitative variables were analyzed by chi square tests. Associations between genotypes or haplotypes and continuous serum lipid levels were analyzed by multivariable linear regression. Differences in serum lipid levels associated with genotypes or haplotypes were considered statistically significant at *p* < 0.0004 or *p* < 0.0005, and G × G or G × E interactions were considered significant at *p* < 0.001 (corresponding to *p* < 0.05 after Bonferroni correction for six SNPs and seven environmental factors). The odds ratios (ORs) and 95% confidence intervals (CIs) were calculated by multiple logistic regression analysis and adjusted by stratified risk factors, including age, sex, cigarette smoking, alcohol consumption, BMI, diabetes, and hypertension. The Hardy-Weinberg equilibrium (HWE), genotypic and haplotypic frequencies, and linkage disequilibrium (LD) described by *D*′ and *R* ^2^ values were calculated by SHEsis online software (http://analysis.bio-x.cn/myAnalysis.php). Generalized multifactor dimensionality reduction (GMDR) online software (http://sourceforge.net/projects/gmdr/) was used to screen optimal SNP-SNP, haplotype-haplotype, G × G, and G × E interaction models. The models meet 10/10 cross-validation consistency (CV) indicating 1,000 permutation tests; both training balance accuracy and test balance accuracy were ≥50%. Furthermore, all permutation tests had *p* < 0.001, suggesting their accuracy predictive value of diseases. An intuitive interactive tree diagram was used to depict the association of each factor in the model, here, the factors were no more than four. All other data visualizations were generated using GraphPad Prism (version 8.0.0).

## Results

### General Characteristics and Clinical Indicators

As shown in Table 1, mean height, weight, SBP, pulse pressure, TG, and the percentage of subjects who smoked cigarettes were higher (*p* ≤ 0.001), whereas mean DBP, HDL-C, ApoA1, the ratio of ApoA1 to ApoB, and the percentage of subjects who consumed alcohol were lower (*p* ≤ 0.004) in CAD than in control groups. Mean height, SBP, DBP, pulse pressure, TG, the percentages of subjects who consumed alcohol or smoked cigarettes, and the prevalence of hypertension were higher (*p* ≤ 0.003), whereas mean HDL-C, ApoA1, and the ratio of ApoA1 to ApoB were lower (*p* ≤ 0.001) in IS than in control groups.

**TABLE 1:** General and biochemical characteristics of the participants.

Parameter	Control	CAD	IS	*P*_CAD_	*P*_IS_
Number	638	622	593	
Male/female	466/172	457/165	428/165	0.863	0.734
Age, years^a^	62.07 ± 12.19	62.34 ± 10.50	62.85 ± 12.17	0.675	0.267
Height, cm	161.76 ± 7.15	164.12 ± 6.91	163.81 ± 7.19	0.000	0.000
Weight, kg	63.41 ± 9.13	65.50 ± 10.34	64.22 ± 10.28	0.000	0.145
Body mass index (BMI), kg/m^2^	24.20 ± 2.93	24.24 ± 3.10	23.87 ± 3.10	0.824	0.050
Cigarette smoking, *n* (%)^b^					
0 cigarette/day	391 (61.30)	360 (57.90)	436 (73.50)		
≤20 cigarettes/day	192 (30.10)	119 (19.10)	84 (14.20)		
>20 cigarettes/day	55 (8.60)	143 (23.0)	73 (12.30)	0.000	0.000
Alcohol consumption, *n* (%)					
0 g/day	365 (57.20)	481 (77.30)	430 (72.52)		
≤25 g/day	34 (5.30)	1 (0.20)	5 (0.84)		
>25 g/day	239 (37.50)	140 (22.5)	158 (26.64)	0.000	0.000
Systolic blood pressure, mmHg	129.18 ± 19.45	133.34 ± 23.57	147.18 ± 22.17	0.001	0.000
Diastolic blood pressure, mmHg	81.46 ± 11.70	79.34 ± 14.36	83.58 ± 13.20	0.004	0.003
Pulse pressure, mmHg	47.80 ± 16.36	54.00 ± 17.64	63.60 ± 17.68	0.000	0.000
Blood glucose, mmol/L	6.28 ± 1.36	6.23 ± 1.87	6.15 ± 1.36	0.551	0.088
Total cholesterol, mmol/L	4.58 ± 0.85	4.56 ± 1.03	4.59 ± 0.97	0.665	0.982
Triglyceride, mmol/L^c^	1.45 ± 0.69	1.59 ± 0.69	1.56 ± 0.73	0.000	0.003
HDL-C, mmol/L	1.57 ± 0.50	1.17 ± 0.28	1.25 ± 0.37	0.000	0.000
LDL-C, mmol/L	2.78 ± 0.54	2.84 ± 0.73	2.83 ± 0.74	0.062	0.161
Apolipoprotein (Apo)A1, g/L	1.33 ± 0.25	1.04 ± 0.21	1.07 ± 0.21	0.000	0.000
ApoB, g/L	0.98 ± 0.21	0.96 ± 0.22	0.98 ± 0.21	0.092	0.050
ApoA1/ApoB	1.42 ± 0.40	1.14 ± 0.32	1.17 ± 0.34	0.000	0.000
BMI >28 kg/m^2^, *n* (%)	63 (9.90)	69 (11.10)	68 (11.50)	0.480	0.365
Hypertension, *n* (%)	113 (17.70)	116 (18.70)	194 (32.70)	0.666	0.000
Age >65 years, *n* (%)	274 (34.50)	253 (40.70)	268 (45.20)	0.414	0.427
Type 2 Diabetes, *n* (%)	139 (21.79)	133 (21.38)	113 (19.06)	0.862	0.235

HDL-C, high-density lipoprotein cholesterol; LDL-C, low-density lipoprotein cholesterol.

a Normally distributed data was described as means ± SD and assessed by Student’s unpaired t-test.

b Qualitative data were assessed using the chi-square test.

c Nonnormally distributed data were described as medians (interquartile range) and assessed using the Wilcoxon-Mann-Whitney test.

### *EHBP1*, *TUBB*, and *WWOX* Genotype and Allele Frequencies

As listed in Table 2, the genotype distribution of the six SNPs in each group met HWE (*p* > 0.05 for all). The genotype and allele frequencies of the rs2278075 SNP were different between the CAD and control groups (*p* < 0.001 for each), and the genotype and allele frequencies of the rs2710642, rs3130685, and rs2278075 SNPs were different between the IS and control groups (*p* < 0.003).

**TABLE 2:** Genotypic and allelic frequencies of the *EHBP1*, *TUBB*, and *WWOX* SNPs in the control and disease groups [*n* (%)].

SNP/genotype/allele	Control (*n* = 638)	CAD (*n* = 622)	IS (*n* = 593)	*P*_CAD_	*P*_IS_
*EHBP1* rs2710642 G > A					
AA	300 (47)	301 (48)	216 (36)		
GA	281 (44)	264 (42)	287 (48)		
GG	57 (9)	57 (9)	90 (15)	0.848	0.000
A	881 (69)	866 (70)	719 (61)		
G	395 (31)	378 (30)	467 (39)	0.756	0.000
*P*_HWE_	0.520	1.000	0.800		
*EHBP1* rs10496099 T > C					
CC	313 (49)	294 (47)	283 (48)		
TC	281 (44)	261 (42)	253 (43)		
TT	44 (7)	67 (11)	57 (10)	0.052	0.222
C	907 (71)	849 (68)	819 (69)		
T	369 (29)	395 (32)	367 (31)	0.122	0.273
*P*_HWE_	0.083	0.460	1.000		
*TUBB* rs3132854 G > T					
GG	427 (67)	422 (68)	406 (68)		
GT	198 (31)	187 (30)	175 (30)		
TT	13 (2)	13 (2)	12 (2)	0.932	0.842
G	1,052 (85)	1,031 (83)	987 (83)		
T	224 (15)	213 (17)	199 (17)	0.774	0.610
*P*_HWE_	0.076	0.160	0.190		
*TUBB* rs3130685 C > T					
CC	184 (29)	167 (27)	95 (16)		
CT	303 (47)	310 (50)	299 (50)		
TT	151 (24)	145 (23)	199 (34)	0.663	0.000
T	605 (47)	600 (48)	697 (59)		
C	671 (53)	644 (52)	489 (41)	0.681	0.000
*P*_HWE_	0.230	1.000	0.350		
*WWOX* rs2222896 A > G					
AA	77 (12)	102 (16)	62 (10)		
AG	312 (49)	299 (48)	249 (42)		
GG	249 (39)	221 (36)	282 (48)	0.073	0.011
G	810 (63)	741 (60)	813 (69)		
A	466 (37)	503 (40)	373 (31)	0.043	0.008
*P*_HWE_	0.200	0.100	0.510		
*WWOX* rs2278075 A > T					
AA	464 (73)	386 (62)	353 (60)		
AT	162 (25)	208 (33)	208 (35)		
TT	12 (2)	28 (5)	32 (5)	0.000	0.000
A	1,090 (85)	980 (79)	914 (77)		
T	186 (15)	264 (21)	272 (23)	0.000	0.000
*P*_HWE_	0.750	1.000	0.820		

EHBP1, EH domain-binding protein 1; TUBB, tubulin beta class I; WWOX, WW domain-containing oxidoreductase; SNP, single nucleotide polymorphism; CAD, coronary artery disease; IS, ischemic stroke; HWE, Hardy-Weinberg equilibrium. Qualitative data were assessed using the chi-square test.

### Association Between the SNPs and CAD/IS

The genetic models of six *EHBP1*, *TUBB*, and *WWOX* SNPs are shown in Table 3. The rs2278075AT/TT genotypes were associated with increased risk of CAD (adjusted OR = 1.63, 95% CI = 1.29–2.07, *p* = 0.0001 for the dominant model). The rs2710642GA/GG and rs2278075AT/TT genotypes were associated with increased risk of IS (adjusted OR = 1.55, 95% CI = 1.23–1.95, *p* = 0.0002 and OR = 1.81, 95% CI = 1.43–2.30, *p* < 0.0001 for the dominant model; respectively), whereas the rs3130685CT/TT genotypes were associated with decreased risk of IS (adjusted OR = 0.61, 95% CI = 0.48–0.79, *p* = 0.0001 for the dominant model).

**TABLE 3:** Associations between the *EHBP1*, *TUBB*, and *WWOX* SNPs and CAD/IS.

SNP/Model	Ref. genotype	Alt. genotype	OR 95% CI_CAD_	*P*_CAD_	OR 95% CI_IS_	*P*_IS_
rs2710642						
Codominant	AA	GA	0.94 (0.74–1.18)		1.42 (1.12–1.80)	
		GG	1.00 (0.67–1.49)	0.850	2.19 (1.51–3.19)	0.0001
Dominant	AA	GA + GG	0.95 (0.76–1.18)	0.630	1.55 (1.23–1.95)	0.0002
Recessive	AA + GA	GG	1.03 (0.70–1.51)	0.890	1.82 (1.28–2.59)	0.0007
Overdominant	AA + GG	GG	1.03 (0.75–1.17)	0.570	1.19 (0.95–1.49)	0.130
Log–additive	AA	GG	0.97 (0.82–1.15)	0.750	1.46 (1.23–1.73)	<0.0001
rs10496099						
Codominant	CC	TC	0.99 (0.78–1.25)		1.00 (0.79–1.26)	
		TT	1.62 (1.07–2.45)	0.052	1.43 (0.94–2.19)	0.220
Dominant	CC	TC + TT	1.07 (0.86–1.34)	0.520	1.05 (0.84–1.32)	0.640
Recessive	CC + TC	TT	1.63 (1.10–2.43)	0.015	1.44 (0.95–2.16)	0.083
Overdominant	CC + TT	TC	0.92 (0.73–1.15)	0.460	0.95 (0.75–1.18)	0.630
Log–additive	CC	TT	1.15 (0.97–1.36)	0.120	1.11 (0.93–1.32)	0.260
rs3132584						
Codominant	GG	GT	0.96 (0.75–1.22)		0.93 (0.73–1.19)	
		TT	1.01 (0.46–2.21)	0.930	0.97 (0.44–2.16)	0.840
Dominant	GG	GT + TT	0.96 (0.76–1.21)	0.730	0.93 (0.73–1.18)	0.560
Recessive	GG + GT	TT	1.03 (0.47–2.23)	0.950	0.99 (0.45–2.19)	0.990
Overdominant	GG + TT	GT	0.96 (0.75–1.21)	0.710	0.93 (0.73–1.17)	0.560
Log–additive	GG	TT	0.97 (0.78–1.20)	0.770	0.94 (0.76–1.17)	0.600
rs3130685						
Codominant	CC	CT	1.13 (0.87–1.47)		0.75 (0.57–0.98)	
		TT	1.06 (0.78–1.44)	0.660	0.39 (0.28–0.54)	<0.0001
Dominant	CC	CT + TT	1.10 (0.86–1.41)	0.430	0.61 (0.48–0.79)	0.0001
Recessive	CC + CT	TT	0.98 (0.76–1.27)	0.880	0.47 (0.36–0.62)	<0.0001
Overdominant	CC + TT	CT	1.03 (0.88–1.20)	0.400	1.12 (0.90–1.41)	0.300
Log–additive	CC	TT	1.03 (0.88–1.20)	0.680	0.63 (0.54–0.75)	<0.0001
rs2222896						
Codominant	GG	AG	1.08 (0.85–1.37)		0.70 (0.56–0.89)	
		AA	1.49 (1.05–2.11)	0.073	0.71 (0.45–1.03)	0.010
Dominant	GG	AG + AA	1.16 (0.92–1.46)	0.200	0.71 (0.56–0.89)	0.0025
Recessive	GG + AG	AA	1.43 (1.04–1.97)	0.028	0.85 (0.60–1.21)	0.370
Overdominant	GG + AA	AG	0.97 (0.78–1.21)	0.770	0.76 (0.60–0.95)	0.015
Log–additive	GG	AA	1.19 (1.01–1.40)	0.041	0.79 (0.67–0.94)	0.075
rs2278075						
Codominant	AA	AT	1.54 (1.21–1.97)		1.69 (1.32–2.16)	
		TT	2.80 (1.41–5.59)	0.0001	3.51 (1.78–6.90)	<0.0001
Dominant	AA	AT + TT	1.63 (1.29–2.07)	0.0001	1.81 (1.43–2.30)	<0.0001
Recessive	AA + AT	TT	2.46 (1.24–4.88)	0.0072	2.98 (1.52–5.83)	0.0008
Overdominant	AA + TT	AT	1.48 (1.16–1.88)	0.0017	1.59 (1.24–2.03)	0.0002
Log–additive	AA	TT	1.58 (1.29–1.95)	<0.0001	1.75 (1.42–2.15)	<0.0001

EHBP1, EH domain-binding protein 1; TUBB, tubulin beta class I; WWOX, WW domain-containing oxidoreductase; SNP, single nucleotide polymorphism; CAD, coronary artery disease; IS, ischemic stroke; Ref., reference; Alt., alternate; OR, odds ratio; CI, confidence interval. The OR and 95% CI were calculated by multiple logistic regression analysis and adjusted by stratified eight risk factors, including age, sex, cigarette smoking, alcohol consumption, BMI, type 2 diabetes mellitus, and hypertension. The P_CAD_ or P_IS_ < 0.00021 was considered statistical significance after the Bonferroni correction (6 SNPs × 5 models × 8 factors).

### Association Between the SNPs and Serum Lipid Levels in Controls

**FIGURE 1:**
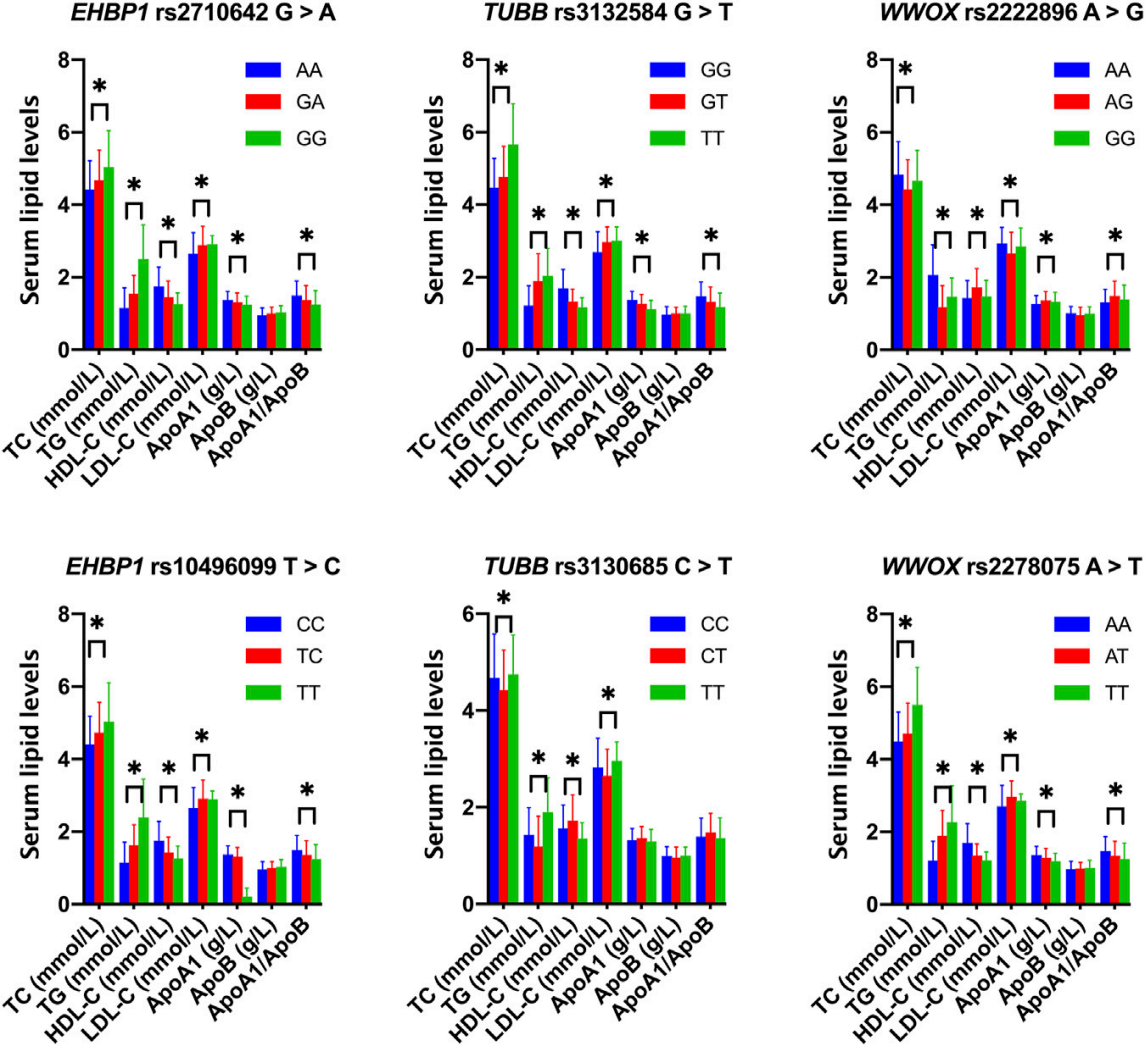
Associations between the 18 *EHBP1*, *TUBB*, and *WWOX* genotypes and serum lipid parameters in the control group. *p < 0.0004 was considered statistical significance after the Bonferroni correction (6 SNPs × 3 genotypes × 7 lipid phenotypes).

As shown in Figure 1, the levels of TC, TG, HDL-C, and LDL-C and the ratio of ApoA1/ApoB in the control group were significantly different among the three genotypes of the six SNPs, and the levels of ApoA1 were also significantly different among the three genotypes of five SNPs, except for rs3130685 SNP (*p* < 0.0004). High levels of TC and TG were associated with the rs2710642G, rs10496099T, rs3132584T, rs3130685T, and rs2222896A alleles; low levels of HDL-C were associated with the rs2710642G, rs10496099T, rs3132584T, and rs2278075T alleles. High levels of LDL-C were associated with the rs2710642G, rs10496099T, and rs3132584T alleles. Low levels of ApoA1 were associated with the rs2710642G, rs10496099T, rs3132584T, and rs3130685T alleles; low ratios of ApoA1/ApoB were associated with the rs2710642G, rs10496099T, and rs3130685T alleles (Table 4). The results were adjusted for confounds including hypertension, obesity, aging, drinking, female sex and diabetes (*p* < 0.0004 for all).

**TABLE 4:** Meaningful associations between the SNPs and serum lipid levels in the control group.

Lipid	SNP Phenotype	Genotype		Beta	*t*	*P*	95% CI of Beta	
		Ref.	Alt.				Lower	Upper
TC	rs2710642	AA	GA	0.144	3.571	<0.0004	0.110	0.380
		AA	GG	0.186	4.594	<0.0004	0.319	0.794
	rs10496099	CC	TC	0.179	4.502	<0.0004	0.172	0.439
		CC	TT	0.156	3.896	<0.0004	0.261	0.791
	rs3132584	GG	GT	0.149	3.840	<0.0004	0.133	0.411
		GG	TT	0.178	4.608	<0.0004	0.634	1.576
	rs3130685	CC	TT	0.148	3.146	0.002	0.107	0.463
	rs2222896	AA	AG	−0.258	−4.105	<0.0004	−0.646	−0.228
TG	rs2710642	AA	GA	0.273	7.922	<0.0004	0.286	0.475
		AA	GG	0.535	15.246	<0.0004	0.360	0.554
	rs10496099	CC	TC	0.327	9.266	<0.0004	0.172	0.439
		CC	TT	0.434	12.240	<0.0004	1.007	1.392
	rs3132584	GG	GT	0.438	12.510	<0.0004	0.553	0.759
		GG	TT	0.150	4.295	<0.0004	0.414	1.111
	rs3130685	CC	TT	0.378	9.322	<0.0004	0.488	0.748
	rs2222896	AA	AG	−0.647	−11.274	<0.0004	−1.053	−0.741
		AA	GG	−0.381	−6.662	<0.0004	−0.701	−0.382
HDL-C	rs2710642	AA	GA	−0.281	−7.555	<0.0004	−0.357	−0.210
		AA	GG	−0.257	−6.875	<0.0004	−0.585	−0.325
	rs10496099	CC	TC	−0.299	−8.135	<0.0004	−0.375	−0.229
	rs10496099	CC	TT	−0.222	−6.017	<0.0004	−0.589	−0.299
HDL-C	rs3132584	GG	GT	−0.334	−9.392	<0.0004	−0.437	−0.286
		GG	TT	−0.137	−3.863	<0.0004	−0.159	−0.247
	rs2278075	AA	AT	−0.312	−8.601	<0.0004	−0.441	−0.277
LDL-C	rs2710642	AA	GA	0.197	5.021	<0.0004	0.131	0.300
		AA	GG	0.117	2.961	0.003	0.076	0.373
	rs10496099	CC	TC	0.215	5.565	<0.0004	0.153	0.319
	rs3132584	GG	GT	0.224	5.977	<0.0004	0.177	0.351
ApoA1	rs2710642	AA	GA	−0.117	−2.922	0.004	−0.098	−0.019
		AA	GG	−0.136	−3.374	0.001	−0.188	−0.050
	rs10496099	CC	TC	−0.125	−3.153	0.002	−0.101	−0.023
		CC	TT	−0.137	−3.425	0.001	−0.211	−0.057
	rs3132584	GG	GT	−0.171	−4.438	<0.0004	−0.131	−0.051
		GG	TT	−0.129	−3.385	0.001	−0.371	−0.099
	rs3130685	CC	TT	−0.178	−3.964	<0.0004	−0.154	−0.052
ApoA1/ApoB	rs2710642	AA	GA	−0.153	−3.786	<0.0004	−0.189	−0.060
		AA	GG	−0.154	−3.792	<0.0004	−0.334	−0.106
	rs10496099	CC	TC	−0.163	−4.071	<0.0004	−0.196	−0.069
		CC	TT	−0.137	−3.408	0.001	−0.348	−0.093
	rs3130685	CC	TT	−0.193	−4.259	<0.0004	−0.269	−0.099

Ref., reference; Alt., alternate. Used multivariable linear regression analysis, p < 0.0004 was considered statistical significance after the Bonferroni correction [6 SNPs × 3 genotypes × 7 factors (age, gender, drinking, smoking, hypertension, diabetes and BMI)].

### Associations Between Haplotypes and CAD/IS, and Serum Lipid Profiles in Controls

**FIGURE 2:**
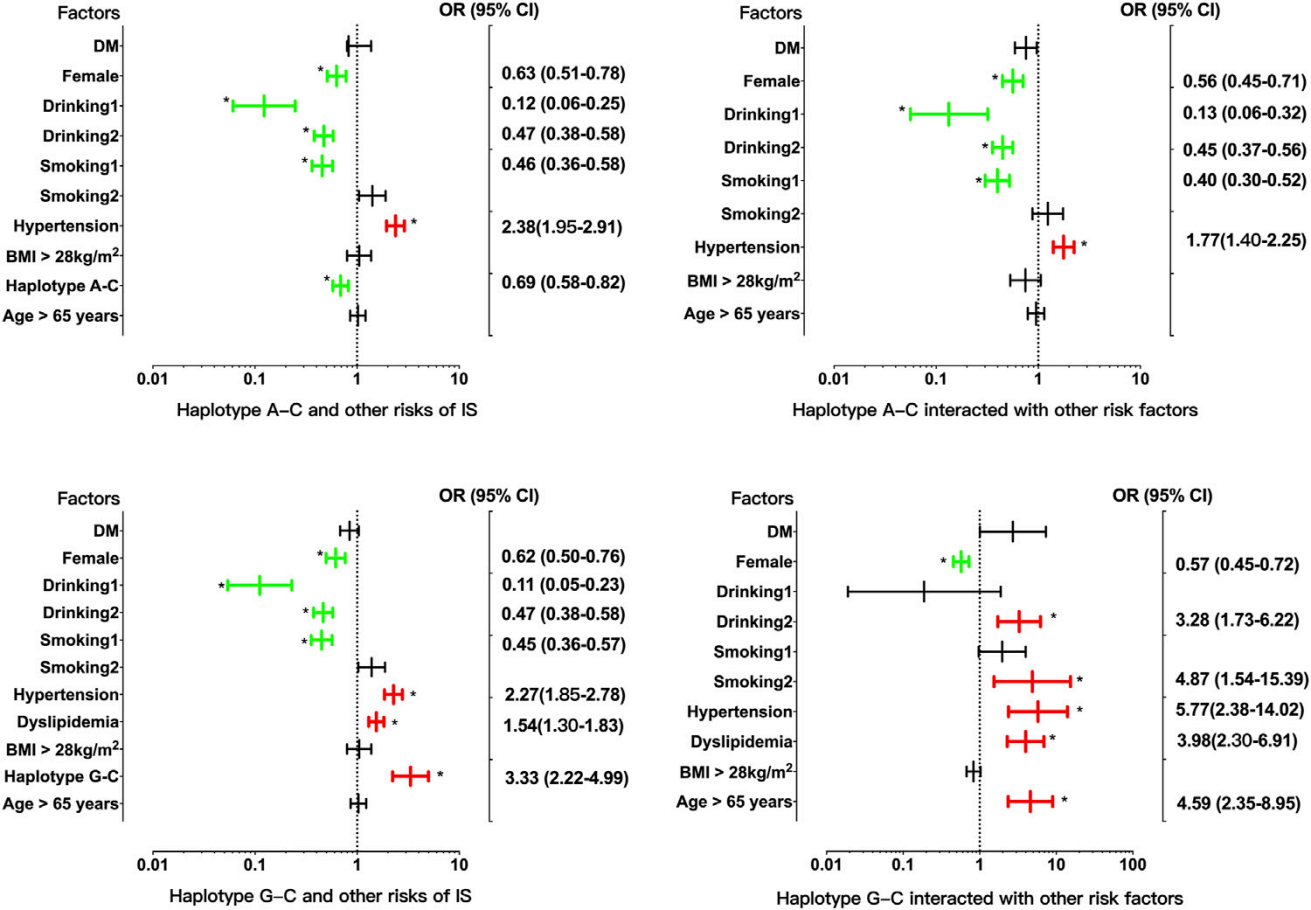
Associations between the *EHBP1* haplotypes, environmental factors, and their interactions on ischemic stroke. *p* < 0.025 was considered statistical significance after the Bonferroni correction for two haplotypes. The red and green colors in the picture mean the increased risk and decreased risk factors, respectively.

There was strong LD between the *EHBP1* rs2710642 and rs10496099 SNPs in the three groups (control, *D'* = 0.951 or *R* ^2^ = 0.821; CAD, *D'* = 1.000 or *R* ^2^ = 0.938; and IS, *D'* = 1.000 or *R* ^2^ = 0.690). Four common haplotypes were detected in the control and disease groups (Table 5). The rs2710642G-rs10496099C haplotype was associated with an increased risk of IS (OR = 2.94, 95% CI = 2.01–4.30, *p* = 7.76 × 10^−9^), whereas the rs2710642A-rs10496099C haplotype was associated with a reduced risk of IS (OR = 0.70, 95% CI = 0.59–0.83, *p* = 2.66 × 10^−5^). Moreover, the two haplotypes interacted with other risk factors for IS (Figure 2) exhibited that, the rs2710642A-rs10496099C haplotype interacted with sex (female), hypertension, and a small amount of smoking or alcohol to decrease the association of IS risk. On the other hand, the rs2710642G-rs10496099C haplotype interacted with sex (female), elderly (age >65 years), a small amount of smoking or alcohol, hypertension and dyslipidemia to increase the association of IS risk (*p* < 0.05 for all). However, there was no association between haplotypes and CAD (*p* > 0.05).

**TABLE 5:** Associations between haplotype frequencies of the two *EHBP1* SNPs and CAD or IS [*n* (frequency)].

Label	Haplotype	Control [*n*(%)]	CAD [*n* (%)]	*χ* ^2^	*P*_CAD_	OR [95% CI] _CAD_	IS [*n* (%)]	*χ* ^2^	*P*_IS_	OR [95% CI] _IS_
H1	A-C	869 (68.10)	849 (68.20)	0.060	0.807	1.02 (0.86–1.21)	719 (60.62)	17.671	2.66 × 10^−5^	0.70 (0.59–0.83)
	A-T	12 (1.00)	17 (1.40)	NA	NA	NA	0 (0.00)	NA	NA	NA
H2	G-C	38 (3.00)	0 (0.00)	NA	NA	NA	100 (8.44)	33.385	7.76 × 10^−9^	2.94 (2.01–4.30)
H3	G-T	357 (27.90)	378 (30.40)	2.001	0.1572	1.13 (0.95–1.35)	367 (30.94)	2.179	0.1399	1.14 (0.96–1.36)

CAD, coronary artery disease; IS, ischemic stroke; A-C, rs2710642A-rs10496099C; A-T, rs2710642A-rs10496099T; G-C, rs2710642G-rs10496099C; G-T, rs2710642G-rs10496099T; n, sample size. NA, not applicable. Lowest frequency thresholds of the four common haplotypes were more than 0.03. Binary logistic regression model was used. p < 0.05 has statistically significant difference.

**FIGURE 3:**
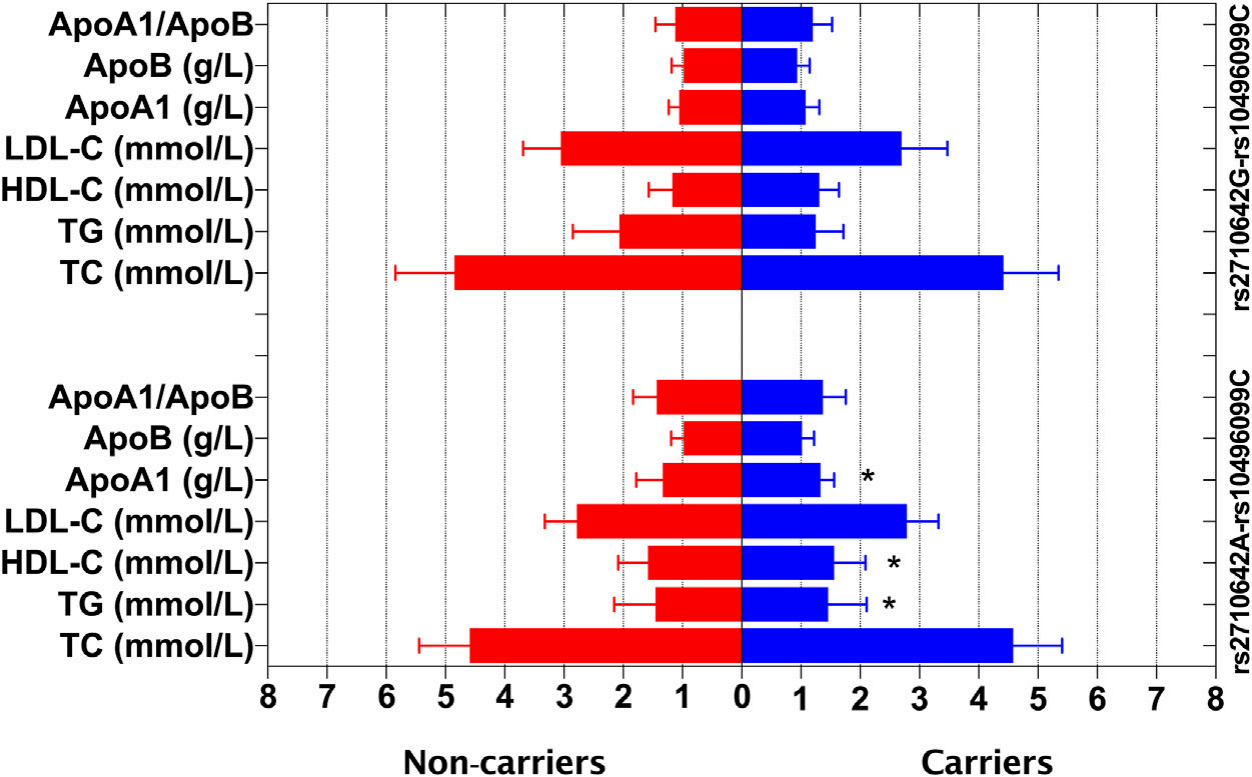
Associations between the *EHBP1* haplotypes and serum lipid levels in the control group. *p < 0.025 was considered statistical significance after the Bonferroni correction for two haplotypes.

In the control group, the levels of TG, HDL-C and ApoA1 were different between the rs2710642A-rs10496099C haplotype carriers and the haplotype noncarriers (*p* < 0.025; Figure 3). After Bonferroni correction, we found that the rs2710642A-rs10496099C haplotype carriers had lower TG and higher HDL-C levels than the haplotype noncarriers (*p* < 0.0005; Table 6). There was no association between the rs2710642G-rs10496099C haplotype and any serum lipid parameters.

**TABLE 6:** Associations between *EHBP1* haplotypes and serum lipid levels in the controls.

Lipid	Haplotype	Carriers	Beta	*t*	*P*	95% CI of Beta	
						Lower	Upper
TC	A-C	Yes/No	−0.044	−1.581	0.114	−0.178	0.019
	G-C	Yes/No	0.002	0.080	0.936	−0.259	−0.281
TG	A-C	Yes/No	−0.151	−5.555	0.000	−0.304	−0.145
	G-C	Yes/No	0.002	0.090	0.929	−0.209	0.230
HDL-C	A-C	Yes/No	0.104	3.957	0.000	0.056	0.167
	G-C	Yes/No	−0.008	−0.297	0.766	−0.175	0.129
LDL-C	A-C	Yes/No	−0.045	−1.671	0.095	−0.114	0.009
	G-C	Yes/No	0.006	0.215	0.830	−0.150	0.187
ApoA1	A-C	Yes/No	0.077	2.844	0.005	0.013	0.069
	G-C	Yes/No	−0.008	−0.297	0.766	−0.089	0.066
ApoB	A-C	Yes/No	−0.001	−0.040	0.968	−0.025	0.024
	G-C	Yes/No	0.031	1.118	0.264	−0.028	0.104
ApoA1/ApoB	A-C	Yes/No	0.051	1.841	0.066	−0.003	0.091
	G-C	Yes/No	−0.028	−1.004	0.316	−0.194	0.063

TC, total cholesterol; TG, triglyceride; HDL-C, high-density lipoprotein cholesterol; LDL-C, low-density lipoprotein cholesterol; ApoA1, apolipoprotein A1; ApoB, apolipoprotein B; A-C, rs2710642A-rs10496099T; G-C, rs2710642G-rs10496099C; CI, confidence interval. p values were calculated by multivariable linear regression. p < 0.0005 indicates a statistically significant difference after Bonferroni correction for 2 haplotypes, 7 lipid profiles and 7 confounding (age, gender, drinking, smoking, hypertension, fasting blood-glucose, and body mass index).

### G × G Interactions on CAD or IS

As listed in Table 7, six interactions of rs2710642-rs10496099-rs3132584-rs3130685-rs2222896-rs2278075 were associated with CAD. Namely, A-C-G-C-A-A (OR = 5.093, 95% CI = 3.897–6.515, *p* = 0.000), A-C-G-T-G-A (OR = 2.562, 95% CI = 2.033–3.229, *p* = 4.03 × 10^−16^), and G-T-T-T-G-T (OR = 1.860, 95% CI = 1.317–2.626, *p* = 3.57 × 10^−6^) were correlated with an increased risk of CAD. On the other hand, A-C-G-C-G-A (OR = 0.229, 95% CI = 0.180–0.291, *p* = 0.000), A-C-G-T-A-A (OR = 0.191, 95% CI = 0.145–0.252, *p* = 0.000), and G-T-G-C-G-A (OR = 0.475, 95% CI = 0.348–0.650, *p* = 2.22 × 10^−6^) were correlated with a decreased risk of CAD. Additionally, four interactions of rs2710642-rs1,0496099-rs3132584-rs3130685-rs2222896-rs2278075 were associated with IS. Namely, A-C-G-T-G-A (OR = 3.046, 95% CI = 2.421–3.834, *p* = 1.56 × 10^−22^) and G-T-G-T-G-A (OR = 6.298, 95% CI = 3.732–10.629, *p* = 6.66 × 10^−15^) were correlated with an augmented risk of IS, while A-C-G-T-A-A (OR = 0.214, 95% CI = 0.164–0.281, *p* = 7.55 × 10^−33^) and G-T-G-C-G-A (OR = 0.484, 95% CI = 0.353–0.664, *p* = 4.90 × 10^−6^) were correlated with a reduced risk of IS.

**TABLE 7:** Frequencies of gene-gene interactions in the control and disease groups [*n* (frequency)].

Label	Gene-gene interaction								*χ* ^*2*^	*P*	OR (95% CI)
	G1	G2	G3	G4	G5	G6	CAD	Control			
I 1	A	C	G	C	A	A	352 (0.283)	82 (0.065)	173.671	0.000	5.093 (3.897–6.515)
I 2	A	C	G	C	G	A	104 (0.084)	316 (0.247)	160.833	0.000	0.229 (0.180–0.291)
I 3	A	C	G	T	A	A	73 (0.059)	271 (0.213)	161.899	0.000	0.191 (0.145–0.252)
I 4	A	C	G	T	G	A	291 (0.234)	120 (0.094)	66.322	4.03 × 10^−16^	2.562 (2.033–3.229)
I 5	G	T	G	C	G	A	66 (0.053)	118 (0.092)	22.444	2.20 × 10^−6^	0.475 (0.348–0.650)
I 6	G	T	T	T	G	T	103 (0.083)	52 (0.041)	12.758	3.57 × 10^−6^	1.860 (1.317–2.626)
							**IS**	**Control**			
I 1	A	C	G	C	A	A	72 (0.060)	82 (0.065)	1.676	0.195	0.805 (0.580–1.118)
I 3	A	C	G	T	A	A	77 (0.065)	271 (0.213)	142.684	7.55 × 10^−33^	0.214 (0.164–0.281)
I 4	A	C	G	T	G	A	316 (0.266)	120 (0.094)	95.527	1.56 × 10^−22^	3.046 (2.421–3.834)
I 5	G	T	G	C	G	A	64 (0.054)	118 (0.092)	20.904	4.90 × 10^−6^	0.484 (0.353–0.664)
I 7	G	T	G	T	G	A	103 (0.087)	17 (0.013)	60.787	6.66 × 10^−15^	6.298 (3.732–10.629)

G1, EHBP1 rs2710642 G > A; G2, EHBP1 rs10496099 T > C; G3, TUBB rs3132584 G > T; G4, TUBB rs3130685 C > T; G5, WWOX rs2222896 A > G; G6, WWOX rs2278075 A > T; I, interaction. Lowest frequency thresholds of gene-gene interaction were more than 0.03.

### Different Interaction Models on CAD or IS

Using GMDR, we screened several models of SNP-SNP, haplotype-haplotype, G × G, and G × E interactions on the risk of CAD and IS, respectively. Nine optimal models (CV constancy of 10 of 10, balanced accuracy test ≥50.28%, and permutation test *p* < 0.001 for all) were significantly associated with CAD (Table 8). They were rs3130685-rs2222896, rs3130685-rs2222896-rs2278075, rs3130685-rs2222896-rs2278075-rs2710642, rs3130685-rs2222896-rs2278075-rs2710642-rs3132584, rs3130685-rs2222896-rs2278075-rs2710642-rs3132584-rs10496099, rs3130685-rs2222896-diabetes, rs3130685-rs2222896-drinking, H1 (rs2710642A-rs10496099C)-H3 (rs2710642G-rs10496099T)-diabetes, and I5 (G-T-G-C-G-A)-I6 (G-T-T-T-G-T)-drinking.

**TABLE 8:** Different types of interaction models related to CAD and IS.

Interactive model	Training bal. acc.	Testing bal. acc.	CV consistency	Sign test *P*	Permutation test *P*
**CAD**					
SNP-SNP					
A-B	0.7184	0.7185	10/10	0.001	<0.0001
A-B-C	0.7398	0.7332	10/10	0.001	<0.0001
A-B-C-D	0.7657	0.7600	10/10	0.001	<0.0001
A-B-C-D-E	0.7792	0.7733	10/10	0.001	<0.0001
A-B-C-D-E-F	0.7886	0.7787	10/10	0.001	<0.0001
SNP-environment					
A-B-G	0.7547	0.7474	10/10	0.001	<0.0001
A-B-H	0.7245	0.7110	10/10	0.001	<0.0001
C-D-G	0.6357	0.6270	10/10	0.004	0.971
Haplotype–haplotype					
H1-H3	0.5137	0.5028	10/10	0.187	0.398
Haplotype-environment					
H1-G-I	0.5754	0.5688	10/10	<0.0001	0.325
H1-H3-G	0.5781	0.5781	10/10	<0.0001	<0.0001
Gene-gene					
I4-I5	0.5381	0.5234	10/10	0.404	0.027
I2-I4-I5	0.5417	0.5377	10/10	0.217	0.027
Gene-environment					
I5-I6-H	0.6302	0.6268	10/10	<0.0001	<0.0001
**IS**					
SNP-SNP					
C-D	0.8297	0.8297	10/10	0.001	<0.0001
A-B-C-D	0.9486	0.9411	10/10	0.001	<0.0001
A-B-C-D-E	0.9657	0.9601	10/10	0.001	<0.0001
A-B-C-D-E-F	0.9672	0.9627	10/10	0.001	<0.0001
SNP-environment					
C-D-J	0.8297	0.8289	10/10	0.001	<0.0001
Haplotype–haplotype					
H1-H2-H3	0.5421	0.5421	10/10	<0.0001	<0.0001
Haplotype-environment					
H1-H2-H	0.5855	0.5855	10/10	<0.0001	<0.0001
H3-G-K	0.5493	0.5462	10/10	<0.0001	<0.0001
Gene-gene					
I3-I4	0.5447	0.5447	10/10	<0.0001	<0.0001
I3-I4-I5	0.5770	0.5770	10/10	<0.0001	<0.0001
I1-I3-I4-I5	0.5827	0.5827	10/10	<0.0001	<0.0001

A = rs3130685, B = rs2222896, C = rs2278075, D = rs2710642, E = rs3132584, F = rs10496099, G = diabetes mellitus, H = drinking, I = female, J = hypertension, K = age >65 years; Bal. Acc., balanced accuracy; CV, cross-validation. H1, haplotype A-C; H3, haplotype G-T; I1, A-C-G-C-A-A; I3, A-C-G-T-A-A; I4, A-C-G-T-G-A; I5, G-T-G-C-G-A. Sign test p < 0.05 indicates a statistically significant different, and permutation test p < 0.001 has a statistically significant difference after Bonferroni correction 6 SNPs and 7 confounding (age, gender, drinking, smoking, hypertension, fasting blood-glucose, and body mass index).

Eleven interaction models (CV constancy of 10 of 10, balanced accuracy test ≥54.21%, and permutation test *p* < 0.001 for all) were significantly related to IS (Table 8). They were rs2278075-rs2710642, rs3130685-rs2222896-rs2278075-rs2710642, rs3130685-rs2222896-rs2278075-rs2710642-rs3132584, rs3130685-rs2222896-rs2278075-rs2710642-rs3132584-rs10496099, rs2278075-rs2710642-hypertension, H1-H2 (rs2710642G-rs10496099C)-H3, H1-H2-drinking, H3-diabetes-aging, I1 (A-C-G-C-A-A)-I3 (A-C-G-T-A-A)-I4 (A-C-G-T-G-A)-I5, I3-I4-I5, and I3-I4.

**FIGURE 4:**
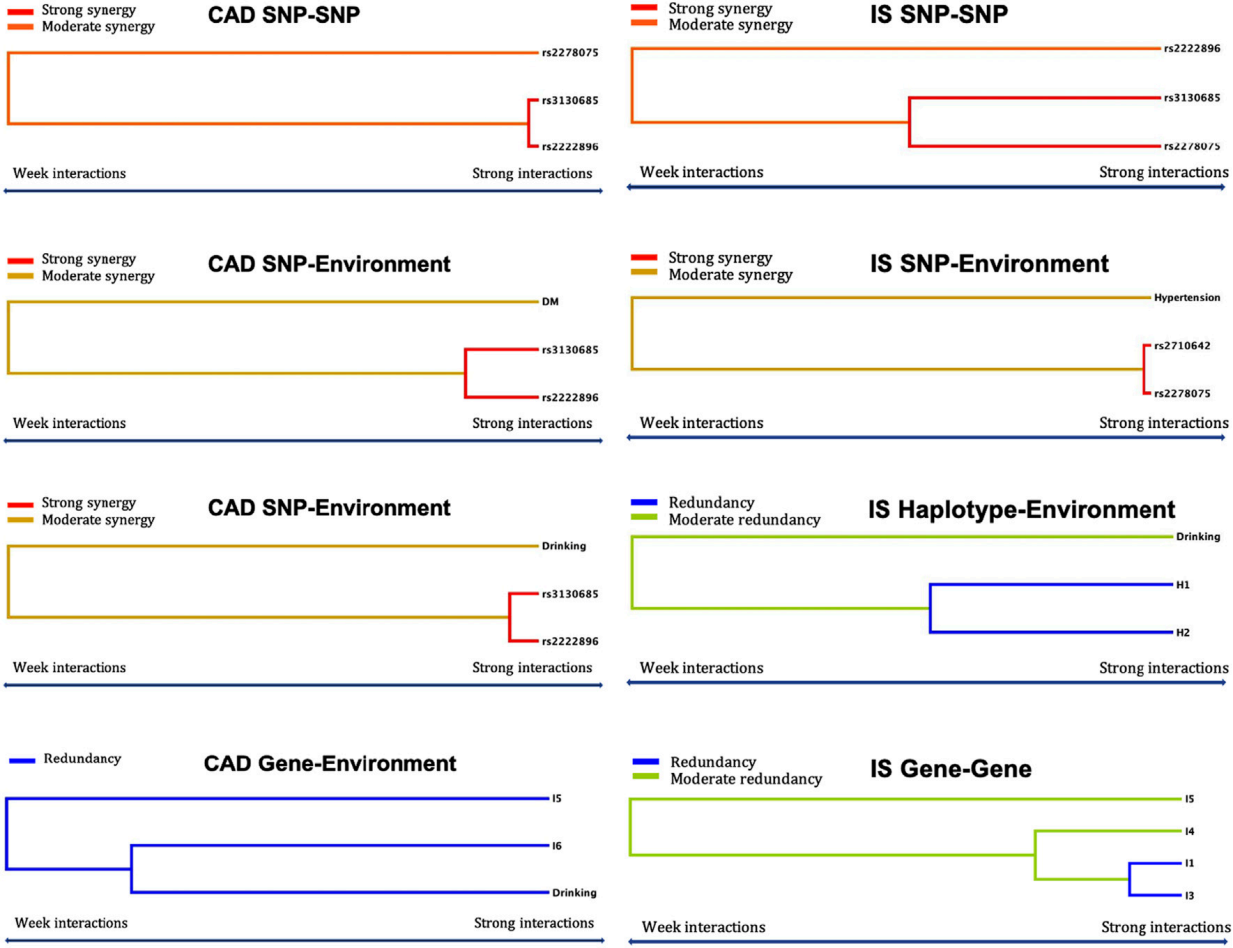
Optimal interactions affecting diseases shown in a dendrogram. Elements that interact strongly with each other appear close together in the leaves of the tree, while elements that interact weakly appear far from each other. The yellow, orange and red colors indicate synergy effect between two factors, and blue and green colors indicate redundancy effect between two factors. The darker the color, the stronger the effect.

Logistic regression analysis was used to further verify the association between the interactions and CAD or IS (Table 9), and the interactive tree diagram (Figure 4) directly exhibited the interaction between risk factors. In the CAD group, subjects carrying H1 and diabetes had higher risk of CAD than those not carrying H1 and diabetes (OR = 1.528, 95% CI = 1.271–1.970, *p* < 0.001), and those drinking but not carrying G-T-T-T-G-T (I6) had lower risk of CAD than those carrying I6 but not drinking (OR = 0.285, 95% CI = 0.234–0.348, *p* < 0.001). In the IS group, compared to those carrying the rs3130685CC or rs2222896AA genotypes, subjects carrying the rs3130685CT + TT and rs2222896AG + GG genotypes were more likely to have a high risk of IS (OR = 2.025, 95% CI = 1.580–2.596, *p* < 0.001). Compared to those carrying the rs2710642GA + GG and rs2278075AA genotypes, subjects carrying the rs2710642AA and rs2278075AT + TT genotypes had an increased risk of IS (OR = 8.740, 95% CI = 5.935–12.871, *p* < 0.001). Compared to those carrying the rs2710642AA and rs2278075 TT genotypes, subjects carrying the rs2710642GA + GG and the rs2278075AT + TT genotypes had an increased risk of IS (OR = 4.723, 95% CI = 3.661–6.095, *p* < 0.001). In addition, compared to those not carrying H1 and not drinking, subjects carrying H1 and drinking had lower risk of IS (OR = 0.463, 95% CI = 0.364–0.589, *p* < 0.001), and compared to those not drinking but carrying H2, subjects drinking but not carrying H2 had lower risk of IS (OR = 0.435, 95% CI = 0.353–0.535, *p* < 0.001).

**TABLE 9:** Risk of CAD or IS with different types of interactions.

Variable 1	Variable 2	OR (95% CI)	*P*
**CAD**			
SNP-SNP interaction			
rs3130685	rs2222896		
CC	AA	1	–
CT + TT	AG + GG	1.293 (1.023–1.635)	0.032
CT + TT	AA	1	–
CC	AG + GG	1.088 (0.863–1.415)	0.533
Haplotype-environment interaction			
H1	DM		
Non-carriers	No	1	–
Carriers	Yes	1.528 (1.271–1.970)	0.000
Non-carriers	Yes	1	–
Carriers	No	0.825 (0.700–0.972)	0.022
Gene-gene interaction			
I2	I5		
Non-carriers	Carriers	1	–
Carriers	Non-carriers	0.875 (0.671–1.141)	0.325
Carriers	Non-carriers	1	–
Non-carriers	Carriers	0.611 (0.446–0.837)	0.002
Gene-environment interaction			
I6	Drinking		
Non-carriers	No	1	–
Carriers	Yes	1.294 (0.288–5.810)	0.736
Carriers	No	1	–
Non-carriers	Yes	0.285 (0.234–0.348)	0.000
**IS**			
SNP-SNP interaction			
rs3130685	rs2278075		
CC	AA	1	–
CT + TT	AT + TT	1.212 (0.939–1.564)	0.140
CT + TT	AA	1	–
CC	AT + TT	0.852 (0.503–1.443)	0.551
rs3130685	rs2222896		
CC	AA	1	–
CT + TT	AG + GG	2.025 (1.580–2.596)	0.000
CT + TT	AA	1	–
CC	AG + GG	0.613 (0.459–0.818)	0.001
rs2710642	rs2278075		
AA	AA	1	–
GA + GG	AT + TT	4.723 (3.661–6.095)	0.000
GA + GG	AA	1	–
AA	AT + TT	8.740 (5.935–12.871)	0.000
Haplotype-environment interaction			
H1	Drinking		
Non-carriers	No	1	–
Carriers	Yes	0.463 (0.364–0.589)	0.000
Carriers	No	1	
Non-carriers	Yes	0.606 (0.447–0.822)	0.001
H2	Drinking		
Non-carriers	No	1	–
Carriers	Yes	1.314 (0.634–2.721)	0.463
Carriers	No	1	–
Non-carriers	Yes	0.435 (0.353–0.535)	0.000

H1, haplotype rs2710642A-rs10496099C; H2, haplotype rs2710642G-rs10496099C; I2, rs2710642A-rs10496099C-rs3132584G-rs3130685C-rs2222896G-rs2278075A; I5, rs2710642G-rs10496099T-rs3132584C-rs3130685C-rs2222896G-rs2278075A; I6, rs2710642G-rs10496099T-rs3132584T-rs313068T5-rs2222896G-rs2278075T; OR, odds ratio; CI, confidence interval. Different types of interactions were analyzed by logistic regression. p < 0.001 indicates a statistically significant difference after Bonferroni correction and adjusting for six SNPs and seven confound factors.

## Discussion

The principal findings of the present study are as follows: ① The rs2278075T allele was associated with an increased risk of CAD. The rs2278075T and rs2710642G alleles were associated with an increased risk, while the rs3130685T allele with a reduced risk of IS. ② The rs2710642G-rs10496099C haplotype was correlated with an increased risk of IS, but the rs2710642A-rs10496099C haplotype was correlated with a decreased risk of IS. ③ Serum lipid phenotypes of rs2278075T, rs2710642G and rs3130685T allele carriers were associated with dyslipidemia, and the haplotype of rs2710642A-rs10496099C was associated with beneficial lipid profiles in the control group. ④ The SNPs of rs2278075, rs2710642 and rs3130685 might be associated with CAD or IS by modifying the serum lipid profiles.

This study found that the MAF of rs2278075 was different between the CAD and control groups, and the MAFs of rs2710642, rs3130685, and rs2278075 were different between the IS and control groups. Moreover, subjects carrying rs2278075TT had an association of a 1.58-fold risk of CAD to those carrying rs2278075AA. This was consistent with a previous GWAS in Caucasian participants (Joseph et al., 2017), and another GWAS reported that smokers carrying *WWOX* were related to coronary artery calcification (Li et al., 2016). These can be explained by the function of *WWOX* in regulating lipoprotein metabolism and lipid homeostasis. In addition, carrying the rs2278075T and rs2710642G alleles were associated with increased risks of IS, while carrying the rs3130685T allele was associated with a reduced risk of IS. We speculate that the rs2278075, rs2710642, and rs3130685 SNPs played a role in regulating blood lipid profiles to change risk of IS. Furthermore, study on the synergistic effects of these SNPs showed that the rs2710642A-rs10496099C-rs3132584G-rs3130685C-rs2222896A-rs2278075A interaction was associated with an increased risk of CAD, and the rs2710642A-rs10496099C-rs3132584G-rs3130685T-rs2222896A-rs2278075A and rs2710642A-rs10496099C-rs3132584G-rs3130685T-rs2222896G-rs2278075A interactions reduced and increased the risk of CAD and IS, respectively. The rs2710642A-rs10496099C-rs3132584G-rs3130685C-rs2222896G-rs2278075A and rs2710642G-rs10496099T-rs3132584C-rs3130685C-rs2222896G-rs2278075A interactions were associated with a reduced risk of CAD, and the rs2710642G-rs10496099T-rs3132584T-rs3130685T-rs2222896G-rs2278075T interaction was associated with an increased risk of CAD. The rs2710642G-rs10496099T-rs3132584G-rs3130685C-rs2222896G-rs2278075A and rs2710642G-rs10496099T-rs3132584G-rs3130685T-rs2222896G-rs2278075A interactions were associated with reduced and increased IS risk, respectively. Many studies have identified that haplotypes have a more significant impact on phenotype than a single SNP (Liu et al., 2020), and the association between haplotypes and phenotypes is more helpful for understanding local ancestral genome information and population genetic structure (Locke et al., 2015). There was strong LD (*D*' > 0.8 or *R* ^2^ > 0.5) between the *EHBP1* rs2710642 and rs10496099 SNPs in the three groups. We found that the rs2710642A-rs10496099C haplotype reduced the risk of IS by 0.7 times, and rs2710642G-rs10496099C haplotype increased the risk of IS by 2.94 times. These results indicate that either coeffects or haplotypes of SNPs have a better predictive effect than any single SNP in the disease risk model of CAD or IS.

Previous studies reported that the rs2710642A and rs3130685T alleles were associated with LDL-C in European, Japanese and the Maonan population in China, and the rs2278075T allele was correlated with CAD in Caucasian (Kathiresan et al., 2007; Giagtzoglou et al., 2013; Joseph et al., 2017). This study observed that in the control group, high levels of TC and TG were associated with the rs2710642G and rs3130685T alleles, and high levels of LDL-C were associated with the rs2710642G allele. However, low levels of HDL-C were associated with rs2278075T allele. These findings suggest that the association of rs2710642, rs3130685 and rs2278075 SNPs with CAD and IS may be achieved by altering blood lipid levels. According to information from the International 1,000 Genomes database (https://www.ncbi.nlm.nih.gov/variation/tools/1000genomes/), the genotypic frequencies and MAFs of the SNPs in Han Chinese individuals in Beijing, China (CHB) were as follows: rs2278075AA 68.3%, AT 19.5%, TT 12.2%, and T 22.0%; rs2710642AA 36.6%, AG 56.1%, GG 7.3% and G 35.4%; and rs3130685CC 20.0%, CT 51.1%, TT 28.9%, and C 45.6%. It exhibited that the mutations and minor alleles of these SNPs were similar between the CHB and Guangxi Han population. Therefore, these SNPs might be associated with an aggravate risk of CAD or IS in CHB population.

The interactions between *EHBP1*, *TUBB*, and *WWOX* and G × E were involved in the pathogenesis of CAD and IS. The interaction of rs2710642 and rs2278075 SNPs was associated with higher risk of IS. The results also showed that the haplotype of rs2710642A-rs10496099C or rs2710642G-rs10496099C interacted with some factors (female sex, few cigarettes, and drinking, especially a small amount of alcohol) to significantly reduce the risk of IS. Some studies have shown that the level of sex hormones and complement of sex chromosomes determined sex differences in CVD patients. Many lifestyles and environmental factors (such as smoking, drinking, and diet) were associated with CVD in gender-specific ways. Differences in sex hormone-dependent receptors that control endothelial function may lead to increased CVD and IS risks in elderly men and postmenopausal women and a slightly lower risk of CVD and IS in premenopausal women (O’Keefe et al., 2018; Owolabi et al., 2018; Polfus et al., 2013). Our study also found that female sex factor was independent or synergistic with lipid-related genes to modulate the risk of IS. The synergy between the haplotype H2 and hypertension and dyslipidemia significantly facilitated the risk of IS. In addition, this study screened the following optimal models to predict the risk of CAD or IS by the GMDR method and binary logistic regression. The interactions of rs3130685-rs2222896-rs2278075, rs3130685-rs2222896-diabetes, and rs3130685-rs2222896-drinking increased the risk of CAD, whereas those of I5-I6-drinking decreased the risk of CAD. The rs3130685-rs2278075-rs2222896 and rs2710642-rs2278075-hypertension interactions strengthened the risk of IS, while H1-H2-drinking and I1-I3-I4-I5 interactions weakened the risk of IS. Many studies have reported that minimal or moderate drinking (≤1 drink/day for women, ≤ 1-2 drink/day for men) is beneficial to protect arteries (Saez et al., 2010; Simino et al., 2013; Sferra et al., 2020). A small amount of red wine before or during dinner could improve the prognosis of CVD (Stanhewicz et al., 2018). The results might be attributed to the fact that grapes are rich in resveratrol, which reduces glutathione peroxidase and serum IL6 levels and has antioxidant and anti-inflammatory activities (Stewart et al., 2019). In this study, drinking and minimal drinking reduced the risk of IS and antagonized the effects of H1 and H2 to reduce IS risk. The explanation might be that the daily alcohol consumption of the subjects in the CAD and IS groups was generally low, or the alcohol content was relatively low. Moreover, this study observed that there was a redundancy effect of the drinking and I6 and I5 interaction in which the drinking factor played a dominant protective effect; thus, the coeffect of I5-I6-drinking reduced the risk of CAD. However, the coeffect of rs3130685-rs2222896-drinking increased CAD risk, here, the mutation of genes was the dominant factor for increasing risk of CAD. Smoking is an independent risk factor for accelerating atherosclerosis (Taskinen and Borén, 2015; Voigt et al., 2020). However, in this study, it showed that minimal smoking independently reduced the risk of IS, and the synergy of minimal smoking with haplotype H1 significantly reduced the risk of IS. Also, minimal smoking also antagonized the risk of IS caused by carrying the haplotype H2. This may be because minimal smoking reduced the psychological pressure of some patients, changing the risk of IS. Also, other protective factors overlapped with minimal smoking, which could mask its adverse effects. It may also be because there were too few subjects with minimal smoking in the IS group, leading to bias in this result. Therefore, a larger sample is needed to replicate the assessment to evaluate the accuracy of this result. Certainly, we have hardly found that minimal smoking has a similar effect on CAD. Hypertension, diabetes and dyslipidemia are independent and traditional risk factors for CVD and cerebrovascular disease (Willer et al., 2013; Wood et al., 2018). Clinical studies have found that with a lower level of normal blood pressure at baseline, hypertension will increase the risk of CAD and IS to a greater extent (Yamada et al., 2019). This study observed that diabetes enhanced the synergistic effect of the rs3130685-rs2222896 interaction, increasing the risk of CAD, and hypertension enhanced the synergistic effect of the rs2710642-rs2278075 interaction, increasing the risk of IS. These results suggest that the interaction of G × E was the risk of CAD and IS. To improve prognosis, intervention for these patients should be individualized, starting with environmental factors or treatments targeting lipid-related genes and their pathways.

Some deficiencies in this study cannot be ignored. First, it did not investigate the dietary habits of the subjects in the three groups and did not analyze the effect of diet as an environmental factor. Second, we did not record the types of antihypertensive or hypoglycemic drugs taken, which cannot be ruled out because of the effect of these drugs on blood lipid levels. Third, there was no resequencing of gene expression after treatment and no follow-up of the prognosis of susceptible gene carriers in the control group. Fourth, a larger cohort sample is needed to verify whether the study has selection bias. Finally, some CAD and IS patients were taking some drugs that may affect serum lipid profiles. Therefore, we could not determine the association between the SNPs and serum lipid levels in CAD and IS groups.

## Conclusion

This study illustrated the associations among *EHBP1*, *TUBB*, and *WWOX* SNPs, G × G and G × E, and CAD and IS in the Guangxi Han population. We found that the *WWOX* rs2278075T allele was associated with the risk of CAD and IS, and *EHBP1* rs2710642 and *TUBB* rs3130685 SNPs might be correlated with IS risk by regulating serum lipid profiles. The haplotypes of *EHBP1* rs2710642 and rs10496099 were more predictive of IS risk than a single SNP. The interactions of G × G and G × E, such as female sex, drinking, smoking, hypertension and diabetes, may alter the association between single risk factor and CAD or IS.

## Data Availability

The data presented in the study have been included in the article/Supplementary Material (the genotypic data), further inquiries can be directed to the corresponding author.
